# Advancing genomics for waterborne pathogen surveillance in Australia

**DOI:** 10.1016/j.lanwpc.2026.101828

**Published:** 2026-03-10

**Authors:** Anson V. Koehler, Marielle Babineau, Karolina Mercoulia, Norelle L. Sherry, Torsten Seemann, Tuyet Hoang, Bill C.H. Chang, Benjamin P. Howden, Robin B. Gasser

**Affiliations:** a The University of Melbourne, Parkville, Victoria, 3010, Australia; b Centre for Pathogen Genomics, The University of Melbourne, Parkville, Victoria, 3010, Australia

**Keywords:** Waterborne pathogen, *Cryptosporidium*, Cryptosporidiosis, “Genotyping”, Genomics, Bioinformatics, National surveillance platform, Climate change, Drug resistance, One Health

## Abstract

Waterborne pathogens, particularly *Cryptosporidium*, pose a growing public-health risk in Australia and globally. Cryptosporidiosis notifications have increased markedly in recent years across multiple countries, driven by greater recreational water exposure, human mobility and climate variability. Climate change, including rising temperatures and extreme weather events, is expected to intensify transmission. Emerging drug resistance in some parasitic pathogens underscores the need for high-resolution surveillance. Despite its status as a nationally notifiable disease, cryptosporidiosis surveillance in Australia relies largely on conventional diagnostic methods that lack sufficient resolution for outbreak detection and source attribution. This Viewpoint examines the rationale for a national genomics-informatics platform integrating whole-genome sequencing, bioinformatics and analytics to modernise surveillance. Drawing on international experience, we outline how genomics can support outbreak tracking and transmission mapping. While bacterial and viral pathogen surveillance has advanced under national genomic initiatives, eukaryotic pathogens remain underrepresented. The proposed platform would leverage existing infrastructure to strengthen responses.

## Introduction—the need for genomics in waterborne disease surveillance

Waterborne pathogens continue to pose major challenges to public health, agriculture and environmental management.^1^ Among them, *Cryptosporidium* is a major protozoan parasite responsible for gastrointestinal illness in humans and animals.^2^^,^^3^ Its resilience to chlorine disinfection, persistence in water supplies and low infectious dose make it a frequent cause of outbreaks linked to drinking water, recreational sources and agricultural run-off. The World Health Organization (WHO) recognises cryptosporidiosis as a global health priority, particularly in low-resource settings where waterborne infections cause high morbidity and mortality, especially among children, immunocompromised individuals and the elderly.^4^, ^5^, ^6^ Ensuring access to safe water and effective pathogen surveillance aligns with the United Nations Sustainable Development Goals (SDGs),^7^ particularly SDG 3 (Good Health and Well-being) and SDG 6 (Clean Water and Sanitation), which emphasise improved water quality, sanitation and disease prevention.^8^

In Australia, cryptosporidiosis is a nationally notifiable disease,^9^ reflecting its public health significance. Yet there is no dedicated genomics-based surveillance system for *Cryptosporidium* or other reportable protozoa, limiting the country's capacity for real-time outbreak tracking, genotype characterisation and targeted intervention. Surveillance usually relies on microscopy, antigen detection and PCR-based “genotyping”.^10^ These methods, while useful, often miss low-level or mixed-species infections and lack the discriminatory power needed for source attribution. Because *Cryptosporidium* cannot be readily cultured, detection in clinical or environmental samples is frequently constrained by low parasite numbers, delaying effective outbreak responses.

Genomic technologies have transformed pathogen surveillance globally.^11^, ^12^, ^13^, ^14^ Whole-genome sequencing (WGS) and metagenomics now underpin bacterial and viral monitoring, providing high-resolution insights into outbreak dynamics, antimicrobial resistance and pathogen evolution.^15^^,^^16^ Applying these approaches to eukaryotic pathogens such as *Cryptosporidium* could markedly improve species identification, outbreak detection and source tracing.^17^^,^^18^ Integrating genomic, epidemiological and environmental data enables a comprehensive view of transmission pathways and risk factors, leading to more precise interventions.^12^^,^^19^ International models in Europe and North America show that genomics-driven surveillance improves early detection and response,^20^ yet Australia has not implemented an equivalent framework. As a result, *Cryptosporidium* transmission remains poorly characterised, and responses are largely reactive. Given the rising global burden of waterborne disease,^21^ adopting genomics for surveillance is now imperative.

Several national initiatives provide a strong foundation. The National Microbial Genomics (NMG) Framework for Public Health 2025–2027,^2^ developed by the Interim Australian Centre for Disease Control (ACDC),^22^ highlights the importance of harmonised, genomics-enabled surveillance and outbreak preparedness. The Australian Pathogen Genomics Program (AusPathoGen), funded by the Medical Research Future Fund (MRFF), is strengthening sequencing capacity and coordination across sectors, creating a natural platform to extend genomics to other eukaryotic pathogens.^23^ This approach aligns with the Australian Drinking Water Guidelines (ADWG),^24^ which emphasise continuous monitoring and risk assessment but have yet to incorporate genomics as a routine surveillance tool. Integrating sequencing and bioinformatics into water-quality management would improve the timeliness and precision of pathogen-risk assessment.

A national genomics-informatics initiative could build on Australia's existing infrastructure, including AusTrakka,^23^^,^^25^^,^^26^ currently focused on bacterial and viral pathogens. Expanding this platform to encompass protozoa would enable coordinated data sharing among public-health agencies, water authorities and research institutions and ensure that genomic intelligence informs evidence-based policy. Integrating genomic epidemiology into waterborne-pathogen surveillance is therefore a logical and necessary step for national preparedness. With the infrastructure and expertise already in place, Australia is well positioned to lead this transformation in the Western Pacific Region.

## The *Cryptosporidium* challenge: an urgent case for genomic tools

A marked rise in cryptosporidiosis cases across Australia^27^, ^28^, ^29^ has renewed attention on the challenges of detecting and managing waterborne parasitic infections. *Cryptosporidium* persists in treated water because of its resistance to chlorination.^30^ Without a genomics-enabled surveillance system, Australia cannot accurately identify infection sources, track transmission routes, or design targeted control and prevention strategies. The steep increase in notifications highlights the need for more sophisticated tools to understand the factors driving transmission and to inform public health responses. Case reports from 2024 illustrate the scale of this increase. In New South Wales, 498 cases were recorded by February—more than five times the five-year average for that period.^31^ Queensland reported 823 cases by early February, with 736 in January alone.^32^ Victoria documented 2349 cases in 2024, a 233% rise from 2023,^33^ and Western Australia reported 800 cases compared with a previous annual mean of 276.^34^ Nationally, 13,166 cases were reported in 2024 *versus* 3715 in 2023,^35^ although the extent to which this reflects true incidence rather than improved reporting remains uncertain.

Multiple interacting factors may underlie this trend. Increased exposure to contaminated recreational water is one plausible driver.^30^^,^^36^^,^^37^ *Cryptosporidium* commonly causes outbreaks linked to swimming pools and water parks, where oocysts persist despite disinfection. High summer temperatures and high-use recreational water venues amplify transmission risk, and public-health alerts in several states have highlighted inadequate hygiene and disinfection practices. Climatic variability may also contribute, with heavy rainfall and flooding introducing microbial contamination into surface waters,^38^^,^^39^ whereas drought increases reliance on suboptimal water sources.^40^ Climate-related shifts in temperature and precipitation may affect parasite survival and distribution, but these effects remain poorly quantified. Human behaviour and hygiene practices are also critical determinants.^10^ Authorities usually advise individuals not to swim for at least two weeks after gastrointestinal illness, yet compliance is inconsistent. Asymptomatic or mildly symptomatic carriers, particularly young children, often continue swimming and sustain transmission within communal pools.^36^ International travel may add complexity, and imported cases from endemic regions have been reported.^41^, ^42^, ^43^ However, the contribution of travel-associated introductions remains uncertain and warrants genomic and epidemiological investigation.

Diagnostic and reporting improvements have likely influenced case numbers. PCR-based tests have increased sensitivity and specificity for *Cryptosporidium* detection, and heightened clinician awareness may have raised notification rates. However, enhanced detection alone cannot explain the magnitude of the increase, indicating that additional epidemiological and environmental factors are operating. Genomics enables direct resolution of the transmission patterns and source relationships underlying these increases. Whole-genome sequencing (WGS) and metagenomic approaches can differentiate *Cryptosporidium* species and genotypes with high precision, enabling identification of transmission networks and outbreak origins.^44^^,^^45^ Genomic data can determine whether surges reflect expansion of a dominant genotype or multiple independent introductions, and can assess whether specific genotypes show enhanced persistence, virulence, or zoonotic potential. Such evidence would directly inform risk assessment and guide intervention design. Translating these capabilities into coordinated national practice requires an integrated surveillance framework that embeds genomics within Australia's public-health system.

Developing a genomics-informed surveillance framework for cryptosporidiosis would align with existing national initiatives, including the NMG Framework and AusPathoGen, both of which emphasise genomics in disease monitoring and outbreak response. Although these programs have focused on bacterial and viral pathogens, extending their scope to eukaryotic organisms such as *Cryptosporidium* would strengthen Australia's overall surveillance framework and enhance national preparedness.

## Lessons from international genomics-based surveillance models

The application of genomics-based surveillance has enhanced the detection and monitoring of *Cryptosporidium* in several countries (Table 1), offering insights into transmission pathways, outbreak sources and public-health interventions. While PCR-based genotyping remains a primary molecular tool for routine surveillance, whole-genome sequencing (WGS) has been introduced in select settings for high-resolution characterisation of *Cryptosporidium* species and genotypes linked to outbreaks. The experiences in the UK, France and the USA provide important models for Australia, where genotyping has been applied but genomic sequencing approaches are not yet part of routine surveillance. In Australia, for example, the work of Professor Una Ryan's group in Perth and our research in Melbourne have been pivotal in defining species- and genotype-level diversity and exploring transmission patterns, thereby establishing a foundation for future genomic integration.

**Table 1:** Select international genomics-based surveillance models for *Cryptosporidium*.

Country	Institution/program	Current practice	WGS integration	Strengths	Limitations
UK	Cryptosporidium Reference Unit (CRU), Swansea^46^	*gp60* subtyping; MLST; national genotyping support^47^^,^^48^	WGS used in outbreak research, not routine surveillance^47^^,^^48^	National coordination; linkage to epidemiological investigations^47^^,^^49^	WGS limited by cost, technical complexity, and DNA yield^47^^,^^48^
France	National Reference Centre for Cryptosporidiosis, Rouen^50^	*gp60* subtyping; MLST; One Health surveillance across sectors^51^, ^52^, ^53^	n/a	n/a	n/a
USA	CryptoNet, Centers for Disease Control and Prevention (CDC)^54^^,^^55^ Academic groups (University of Georgia)^45^^,^^56^, ^57^, ^58^, ^59^, ^60^	National genotyping surveillance using *gp60* and MLST^54^	WGS applied in targeted outbreak resolution^45^^,^^56^^,^^60^	Structured national platform^54^; use of WGS^45^^,^^56^	WGS not yet applied universally; resource-intensive^56^^,^^60^
Australia	Academic groups (Murdoch University, University of Melbourne, WEHI), coordinated via CDGN^44^^,^^61^, ^62^, ^63^, ^64^, ^65^, ^66^	Genotyping (*gp60*, *SSU* rDNA)^62^, ^63^, ^64^, ^65^, ^66^; WGS in select research studies^44^^,^^61^	Not yet integrated into routine public health surveillance^44^^,^^61^^,^^64^	Strong academic and laboratory research base; aligns with NMG and AusTrakka^23^^,^^25^^,^^26^	Absence of national WGS surveillance for eukaryotic pathogens^64^
Canada	National Microbiology Laboratory (NML), PHAC^67^^,^^68^	Routine genotyping^67^^,^^68^	n/a	n/a	n/a
Europe	European Centre for Disease Prevention and Control; EpiPulse^69^	MLST^70^^,^^71^	n/a	n/a	n/a

*Abbreviations:* WGS = whole-genome sequencing; MLST = multilocus sequence typing; *gp60* = glycoprotein 60 gene locus; *SSU* rDNA = small subunit ribosomal DNA; *CDGN* = Communicable Diseases Genomics Network; *NMG* = National Microbial Genomics Framework; AusTrakka = Australian Pathogen Genomics Platform; NML = National Microbiology Laboratory; PHAC = Public Health Agency of Canada; CDC = Centers for Disease Control and Prevention; n/a = not applicable.

In the UK, the Cryptosporidium Reference Unit (CRU, Swansea, UK),^46^ led by Dr Rachel Chalmers, serves as the national reference centre for *Cryptosporidium* diagnostics and epidemiology.^47^ The CRU provides genotyping services for public-health agencies, supporting investigations of isolates from humans, animals and the environment. Among the molecular tools employed—including multiple-locus variable-number tandem-repeat analysis (MLVA),^72^ variable-number tandem-repeat (VNTR)^73^ and the *gp60* subtyping method,^74^ which differentiates strains based on genetic variation in a gene (*gp60*) encoding a key surface glycoprotein has been the most widely used approach for tracing outbreaks and assessing zoonotic potential. This method has successfully linked cases to contaminated drinking water, recreational water and agricultural sources.^75^, ^76^, ^77^, ^78^, ^79^, ^80^, ^81^, ^82^

Although genotyping has been the mainstay of surveillance, the CRU has introduced genomic sequencing for research purposes, particularly in outbreak investigations. Advances in direct sequencing from clinical samples have enabled high-resolution studies without parasite propagation.^41^^,^^48^^,^^83^, ^84^, ^85^ However, WGS is not yet used routinely in public-health investigations, primarily because of cost,^47^ technical complexity and the challenge of extracting sufficient DNA from clinical specimens.^48^^,^^56^^,^^86^^,^^87^ The UK experience demonstrates both the potential of WGS to substantially improve *Cryptosporidium* characterisation during outbreaks and the practical challenges of implementing such a platform at scale.

In France, the National Reference Centre for Cryptosporidiosis in Rouen, led by Professor Loïc Favennec, has played a key role in genotyping-based surveillance and outbreak response. Like the UK, France relies on *gp60* subtyping and multilocus sequence typing (MLST) to differentiate *Cryptosporidium* species and subtypes.^50^ These techniques have been used to define transmission routes in human, veterinary and environmental settings, supporting a One Health approach to surveillance.^50^, ^51^, ^52^, ^53^ More recently, French researchers have applied WGS in specific outbreak studies, providing detailed analyses of strain variation and persistence across reservoirs. As in the UK, however, WGS remains primarily a research tool, with genotyping still forming the basis of routine public-health investigations. France's experience underscores the importance of integrating genomic research with epidemiological investigations to ensure that sequencing technologies translate effectively into public-health practice.

In contrast, the USA has adopted a more structured approach to integrating WGS into cryptosporidiosis surveillance. The Centers for Disease Control and Prevention (CDC) established CryptoNet,^54^ the first national molecular surveillance system for *Cryptosporidium*.^55^ While genotyping remains the primary method for routine case investigations, the CDC has incorporated WGS into outbreak analyses. Through CryptoNet, WGS has been applied to resolve transmission events that genotyping alone could not determine, particularly in outbreaks linked to recreational water, drinking water and foodborne sources. WGS has enabled high-resolution tracing of transmission clusters and improved understanding of outbreak dynamics. This work demonstrates that, while genotyping serves as an effective screening tool, WGS provides critical resolution for precise genomic identification and differentiation of *Cryptosporidium* species and variants. Even in the USA, however, WGS is not yet universally applied to all cryptosporidiosis cases, reflecting the resource-intensive nature of whole-genome analysis.

Genomic-surveillance studies of *Cryptosporidium* across the UK, USA, Australia, Ghana, Tanzania, Madagascar, Gabon, Egypt, China and several EU member states have yielded critical insights into parasite diversity, evolution and transmission.^44^^,^^45^^,^^49^^,^^56^^,^^61^ Advances in population genomics have clarified the roles of recombination, host adaptation and lineage divergence, while also exposing technical and analytical challenges. Together, these studies highlight the power of WGS and metagenomics to resolve cryptic diversity and transmission dynamics. Long-read and telomere-to-telomere assemblies have revealed structural plasticity in *Cryptosporidium parvum*, including subtelomeric gene duplications and expansion of transporter-gene families, providing a high-resolution view of genome organisation.^57^^,^^58^ Other work has traced the emergence and transcontinental spread of outbreak-associated lineages^45^ and shown the impact of recombination between zoonotic and anthroponotic populations at virulence-associated loci.^88^ Key methodological advances—such as hybridisation-based enrichment protocols enabling sequencing directly from clinical stool samples—now support broader public-health application of WGS.^59^ For *Cryptosporidium hominis*, population-scale genomic studies have uncovered deep phylogeographic structuring, particularly in Africa, shaped by recombination and admixture among regional lineages.^61^ Globally, the recognition of two divergent subspecies, *C. hominis hominis* and *C. hominis aquapotentis*, has provided a new framework for understanding transmission and adaptation across socioeconomic and ecological contexts.^44^ These collective findings, synthesised in recent reviews,^56^^,^^89^ establish an empirical and conceptual framework for a genomics-informed surveillance system capable of detecting emerging genotypes, tracking transmission networks and informing targeted responses to cryptosporidiosis.

In Australia, genotyping-based studies have substantially advanced understanding of *Cryptosporidium* transmission,^3^ although WGS has yet to be widely implemented in surveillance. The work of Professor Una Ryan at Murdoch University in Perth has been central to defining *Cryptosporidium* genotypes in humans, livestock and wildlife, clarifying zoonotic transmission risks.^3^^,^^10^^,^^62^^,^^90^, ^91^, ^92^, ^93^, ^94^, ^95^ Similarly, our research in Melbourne has provided important genotypic data supporting epidemiological investigations and waterborne-disease risk assessments.^63^^,^^96^, ^97^, ^98^ While genotyping remains the cornerstone of cryptosporidiosis surveillance in Australia, the potential for WGS to enhance outbreak detection and risk assessment is clear. The experiences of the UK, France and the USA indicate that a phased implementation—beginning with targeted sequencing of outbreak-associated strains—could enable Australia to develop a robust genomic-surveillance system.

## The case for a genomics-informatics platform for *Cryptosporidium* and other eukaryotic pathogens in Australia

Building on the rationale outlined above, Australia is well positioned to advance its waterborne-disease surveillance capabilities through the establishment of a genomics-informatics platform for *Cryptosporidium* and other eukaryotic pathogens, leveraging existing strengths developed for viral and prokaryotic pathogens.

Unlike bacteria, *Cryptosporidium* poses specific challenges for genomic surveillance, including difficulty in culturing the parasite and the low concentration of oocysts in clinical and environmental samples. Despite these challenges, international models in the UK, France and the USA have demonstrated the feasibility of integrating WGS into cryptosporidiosis monitoring (Table 1). In Australia, existing microbial-genomics initiatives provide a strong foundation for expansion into the eukaryotic pathogen area. The National Microbial Genomics (NMG) Framework, AusPathoGen and the Australian CDC all support the integration of genomics into infectious-disease monitoring. Aligning the proposed genomics-informatics platform with these initiatives would enhance coordination, standardisation and long-term sustainability.

These initiatives demonstrate that nationally coordinated, multi-jurisdictional genomic surveillance is operationally feasible within the Australian public-health system. Programs such as AusPathoGen and AusTrakka have successfully integrated federal and state health agencies, public-health laboratories, academic centres and industry partners through shared governance structures, standardised data pipelines and agreed data-sharing frameworks. The proposed *Cryptosporidium* surveillance platform draws directly on these established operating models, adapting proven coordination, workforce and informatics strategies to the specific biological and epidemiological characteristics of eukaryotic pathogens rather than requiring the creation of a new governance framework.

### The need for a national genomics-informatics platform

**Fig. 1:**
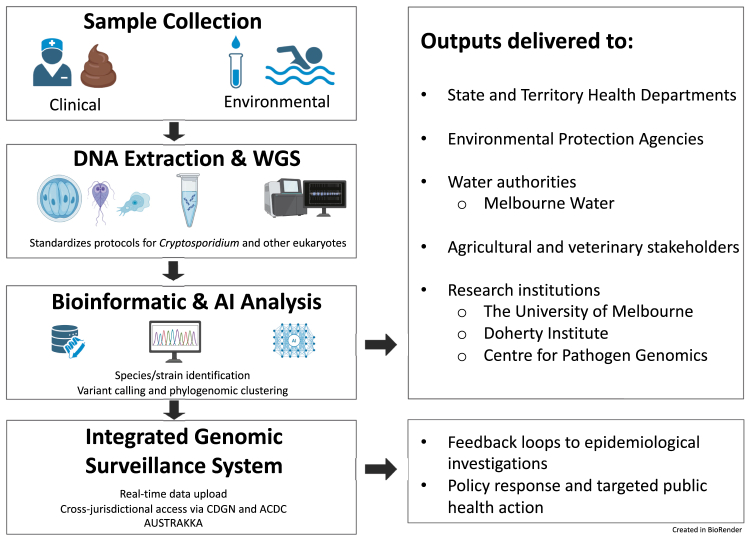
**Framework for a national genomics-informatics platform for *Cryptosporidium* and eukaryotic pathogen surveillance in Australia.** This flowchart illustrates the proposed surveillance architecture, comprising sequential components from clinical and environmental sample collection to real-time data reporting. Samples are processed using standardised protocols for DNA extraction and whole-genome sequencing (WGS), followed by bioinformatics and artificial intelligence (AI)-driven analyses for species/strain identification, variant calling and phylogenomic clustering. Genomic data are uploaded to an integrated national surveillance system, enabling cross-jurisdictional access via AusTrakka with governance supported by the Communicable Diseases Genomics Netowrk (*CDGN*) and Australian Centre for Disease Control (*ACDC*) frameworks. Outputs are delivered to state and territory health departments, environmental protection agencies, water authorities, agricultural and veterinary sectors, and research institutions. The platform supports feedback loops to outbreak investigations and informs policy responses and targeted public health action. This model is aligned with the National Microbial Genomics Framework (*NMGF*) and AusTrakka infrastructure, and is adaptable for broader One Health applications.

A national genomics-informatics platform for cryptosporidiosis surveillance would enhance Australia's capacity to detect outbreaks earlier, understand transmission pathways and identify zoonotic and environmental reservoirs. By integrating genomic, epidemiological and environmental data, the platform would enable more accurate risk assessment and support targeted interventions (Fig. 1). It would also strengthen Australia's broader public-health-genomics strategy. The NMG Framework emphasises nationally coordinated microbial-genomics efforts to ensure that genomic data are accessible and standardised across agencies. Similarly, AusPathoGen—which integrates genomic tools into national infectious-disease surveillance—provides a framework readily extendable to cryptosporidiosis and other parasitic diseases.

In practice, a national genomics-enabled surveillance model would build on existing diagnostic and public-health workflows. Samples would be drawn primarily from routine clinical diagnostics for notified cryptosporidiosis cases, supplemented by outbreak-associated specimens and targeted environmental samples from drinking-water catchments and high-risk recreational water settings. Clinical samples would typically include stool specimens, while environmental sampling would focus on water concentrates collected during periods of increased risk or incident response. Routine molecular detection and genotyping would remain first-line approaches, with escalation to whole-genome sequencing triggered by defined criteria such as case clustering, suspected outbreaks, unusual or emerging genotypes or signals of zoonotic or environmental transmission. Whole-genome sequencing would therefore be applied to a subset rather than all detected cases, with indicative volumes in the order of tens to hundreds of genomes annually at national scale. Incremental costs are expected to be modest relative to established bacterial genomic-surveillance programmes, particularly when existing sequencing platforms, bioinformatics infrastructure and workforce capacity are leveraged, and are likely to decrease further as methods mature and scale increases.

### Key components of the proposed platform

#### Coordinated governance and policy framework

Successful implementation would require collaboration among the ACDC, state and territory health departments, research institutions, the water industry and regulatory agencies. Establishing a national policy framework would standardise data collection, analysis and reporting, while interoperability with existing surveillance systems would ensure that real-time genomic insights inform outbreak investigations and water-safety measures. A centralised data-sharing agreement would facilitate collaboration across jurisdictions.

#### Genomic sequencing and laboratory infrastructure

Expanding WGS capacity for *Cryptosporidium* and other eukaryotic pathogens within the public-health laboratory network would enable comprehensive outbreak tracking. Laboratories across major states and territories could function as regional hubs, building jurisdictional capacity and expertise. Standardised workflows for DNA extraction and sequencing would ensure data reproducibility, a prerequisite for a national genomic-surveillance system.

#### Bioinformatics and data-management systems

A national *Cryptosporidium* genomic database would be essential for storing, analysing and comparing sequences across outbreaks. Integration with existing microbial-genomics databases would allow application of artificial intelligence (AI)-supported analytics to identify genetic diversity and track transmission patterns. Automated data-sharing mechanisms between state health departments, the ACDC and environmental agencies would enable real-time epidemiological investigations and rapid containment.

#### Epidemiological integration and public-health applications

Linking genomic surveillance with epidemiological data would markedly strengthen outbreak-detection and response capacity. Integrating genomic data with environmental sampling, water-quality testing and patient metadata would provide a comprehensive understanding of transmission dynamics. Collaboration with veterinary and agricultural sectors would ensure that zoonotic risks are included in investigations, reinforcing a One Health approach to cryptosporidiosis surveillance.

#### Workforce development and capacity building

Training public-health professionals and epidemiologists in genomic data analysis and interpretation will be critical to sustaining the platform. National and international training programs could incorporate genomic epidemiology into public-health education and laboratory-accreditation schemes, coordinated through laboratories with existing expertise, potentially under the auspices of the Communicable Diseases Genomics Network (CDGN). Developing specialist roles within public-health agencies would further support the interpretation and use of genomic data in outbreak management.

#### Public-health communication and risk assessment

Genomics-based surveillance must be accompanied by clear communication strategies. Translating genomic insights into actionable public-health messages will guide water-quality management, risk communication and community-level interventions. Collaboration among water utilities, regulators, environmental agencies and local governments will ensure that evidence-based policies mitigate waterborne-disease risks.

### Proposed implementation roadmap: a staged approach

A staged approach to implementation would allow for gradual expansion and optimisation, ensuring that genomic surveillance becomes an integral part of Australia's public-health infrastructure.

#### Phase 1

Feasibility and Pilot Studies (Years 1 and 2)—Conduct pilot WGS studies on cryptosporidiosis outbreaks to assess feasibility. Evaluate existing sequencing capacity within public-health laboratories. Develop initial data-sharing agreements.

#### Phase 2

Infrastructure Development and Network Expansion (Years 3 and 4)—Expand WGS capacity across reference laboratories in major states. Establish a national genomic database under the NMG Framework. Implement training programs for public health and laboratory staff.

#### Phase 3

Full National Integration (Years 5 and 6)—Incorporate WGS into routine cryptosporidiosis surveillance. Establish real-time genomic data-sharing mechanisms for outbreak tracking. Conduct ongoing evaluations to refine public-health applications of genomic data.

#### Phase 4

Future Expansion and Long-Term Viability (Year 7 onwards)—For the platform to remain viable long-term, it must be financially sustainable and adaptable to future technological advances. This would require stable funding from government programs, supported by research grants and public-private partnerships. Interoperability with other genomic surveillance programs, ensuring cross-disease applicability. Collaboration with international genomic surveillance networks, strengthening Australia's contribution to global waterborne disease monitoring.

## Impact and future directions

The proposed genomics-informatics platform for cryptosporidiosis surveillance will enhance Australia's capacity to detect, track and respond to waterborne disease outbreaks. Integrating WGS and bioinformatics into public-health monitoring will provide higher resolution for outbreak detection and risk assessment, enabling earlier intervention and more effective responses. Although initially focused on *Cryptosporidium*, the platform will also create a framework for the genomic surveillance of a wide range of other eukaryotic pathogens, including emerging and neglected parasites.^11^^,^^99^ Expanding the platform's scope will contribute to biosecurity efforts, particularly in protecting Australia's borders from the introduction of exotic parasites. By aligning with the NMG Framework, AusPathoGen and the ACDC, this initiative will ensure national coordination in genomic surveillance and reinforce One Health research.

### Public health and biosecurity gains

The ability to rapidly detect and accurately characterise *Cryptosporidium* outbreaks will provide significant public health benefits. A genomics-based approach will enable public-health agencies to detect outbreaks earlier, identify transmission routes more accurately and implement timely control measures.

Genomic surveillance will also strengthen Australia's biosecurity by providing early warnings of novel *Cryptosporidium* strains or other emerging eukaryotic pathogens. The platform will support efforts to protect Australia's borders from the introduction of exotic parasites by distinguishing between locally circulating and imported strains. With increasing global movement of people, animals and goods, ensuring Australia's preparedness against invasive parasitic species is critical. Beyond *Cryptosporidium*, the platform can expand to monitor other protozoan, helminthic and ectoparasitic infections, improving understanding of parasite transmission dynamics, environmental persistence and emerging resistance patterns. This broader application will help integrate genomic surveillance into national One Health strategies, linking human, animal and environmental health data.

### Economic and industry benefits

Cryptosporidiosis and other waterborne disease outbreaks impose substantial economic costs, including healthcare expenditures, productivity losses and expenses associated with outbreak management. A genomics-based surveillance system will enable earlier detection and intervention, reducing these costs and minimising disruption to public health, agriculture and the water sector.

The acquisition of genomic data sets will also benefit agriculture and food safety, particularly in tracking zoonotic *Cryptosporidium* genotypes in livestock and food-production systems. Improved monitoring of parasite contamination in drinking water and irrigation networks will enhance biosecurity for food exports and domestic markets. Investment in genomics and bioinformatics will create new opportunities for biotechnology, diagnostics and environmental-health research. Advances in pathogen genomics, molecular epidemiology and computational modelling will foster public–private partnerships, stimulating innovation in diagnostic tool development and data-driven public-health strategies.

### Scientific and global leadership

Establishing a national genomics-informatics platform will reinforce Australia's position as a leader in environmental pathogen genomics and One Health research. The ability to generate, analyse and share genomic data on *Cryptosporidium* and other eukaryotic pathogens will strengthen global collaborations and international public-health initiatives.

Australia's participation in global genomic-surveillance efforts, including collaborations with the UK's Cryptosporidium Reference Unit, the CDC's CryptoNet and the French National Reference Centre for Cryptosporidiosis, will facilitate comparative genomic studies. The platform will generate standardised genomic data to monitor pathogen distribution, track cross-border transmission and assess the effectiveness of interventions. Beyond cryptosporidiosis, the platform will support research into neglected parasitic diseases, providing new insights into emerging zoonoses and vector-borne infections. Expanding WGS applications to helminths and ectoparasites will further strengthen Australia's contributions to global biosecurity, environmental microbiology and infectious-disease control.

### Future directions and sustainability

For long-term sustainability, the CDGN—on behalf of the ACDC and in collaboration with state health departments and research institutions—should oversee integration of genomic surveillance into public-health infrastructure. Ensuring that genomic data are routinely applied in disease monitoring, outbreak response and border biosecurity measures will be critical to maintaining the platform's impact. Expanding its scope to other eukaryotic parasites should be approached strategically, beginning with pilot projects on high-priority pathogens.

Training programs will be essential to ensure that public-health professionals, bioinformaticians and parasitologists can analyse and interpret genomic data. Moreover, a sustainable funding model will be required to maintain investment in sequencing technology, bioinformatics infrastructure and workforce development. Government research grants, industry partnerships and international collaborations should be leveraged to ensure long-term financial viability.

## Conclusion—the time for action is now

The rising burden of cryptosporidiosis and other waterborne diseases underscores the urgent need for genomics-driven surveillance in Australia. Current approaches, reliant on PCR-based genotyping, provide valuable epidemiological insights but lack the resolution needed for precise outbreak detection, source attribution and risk assessment. Establishing a national genomics-informatics platform for cryptosporidiosis surveillance will modernise Australia's capacity to detect, track and respond to waterborne disease outbreaks. By integrating WGS, bioinformatics, analytics and epidemiological data, this initiative would transform waterborne-disease monitoring, enhance outbreak preparedness and strengthen biosecurity. Aligning with national frameworks such as the NMG Framework, AusPathoGen and the Australian CDC, the platform would enable a shift from reactive to proactive, evidence-based disease management. Automated data-sharing systems and advanced analytics would support real-time outbreak detection, transmission tracking and targeted interventions, ensuring coordinated responses across public-health agencies, research institutions and water-regulatory bodies.

Beyond cryptosporidiosis, the platform provides a scalable model for the genomic surveillance of other neglected parasitic diseases, including protozoa, helminths and ectoparasites. Expanding genomic applications to these pathogens would support One Health collaborations that integrate human, animal and environmental surveillance to mitigate emerging infectious threats. This initiative would also reinforce Australia's biosecurity, protecting national borders from exotic parasites and strengthening global pathogen-surveillance efforts. The economic and industry benefits extend well beyond public health: enhanced food and water safety, improved biosecurity measures and strengthened outbreak-response capabilities would safeguard key sectors such as agriculture, aquaculture and environmental management. Investment in genomic infrastructure, workforce development and cross-sector partnerships would drive innovation in biotechnology, bioinformatics and molecular diagnostics, positioning Australia as a global leader in eukaryotic pathogen genomics.

A phased implementation strategy—beginning with targeted WGS studies and gradually scaling up to full integration—will ensure cost-effective adoption and long-term sustainability. Continued strategic investment and collaboration across government, academia and industry are essential to embed genomic surveillance of eukaryotic pathogens as a core pillar of Australia's public-health and biosecurity framework. The time to act is now. By committing to this initiative, Australia can future-proof public health, strengthen biosecurity and establish itself as a leader in pathogen genomics in the Western Pacific Region. This platform would deliver sustainable, evidence-based water-management strategies for decades to come, safeguarding human, animal and environmental health.

## Contributors

RBG and AVK: Concept, synthesis, writing original draft, writing, reviewing and/or editing. MB, KM, NLS, TS, TH, BCHC, BPH: reviewing or editing.

## Declaration of generative AI and AI-assisted technologies

The authors are responsible for the writing, review and editing of the manuscript. ChatGPT-4o was used solely for formatting or editing of some sections. All content was critically reviewed and approved by the authors.

## Declaration of interests

All authors declare no conflict of interest.

## References

[1] Afonso A.C., Saavedra M.J., Gomes I.B., Simões M., Simões L.C. (2025). Current microbiological challenges in drinking water. J Water Process Eng.

[2] Innes E.A., Chalmers R.M., Wells B., Pawlowic M.C. (2020). A one health approach to tackle cryptosporidiosis. Trends Parasitol.

[3] Ryan U., Zahedi A., Feng Y., Xiao L. (2021). An update on zoonotic *Cryptosporidium* species and genotypes in humans. Animals.

[4] Checkley W., White A.C., Jaganath D. (2015). A review of the global burden, novel diagnostics, therapeutics, and vaccine targets for *Cryptosporidium*. Lancet Infect Dis.

[5] Khalil I.A., Troeger C., Rao P.C. (2018). Morbidity, mortality, and long-term consequences associated with diarrhoea from *Cryptosporidium* infection in children younger than 5 years: a meta-analyses study. Lancet Glob Health.

[6] World Health O (2025). https://iris.who.int/handle/10665/380569.

[7] Affairs UNDoEaS Sustainable Development Goals. https://sdgs.un.org/goals.

[8] Nations U UN-Water. https://www.unwater.org.

[9] NNDSS National notifiable diseases surveillance system. https://www.health.gov.au/our-work/nndss.

[10] Ryan U.M., Feng Y., Fayer R., Xiao L. (2021). Taxonomy and molecular epidemiology of *Cryptosporidium* and *Giardia* - a 50 year perspective (1971-2021). Int J Parasitol.

[11] Gardy J.L., Loman N.J. (2018). Towards a genomics-informed, real-time, global pathogen surveillance system. Nat Rev Genet.

[12] Pronyk P.M., de Alwis R., Rockett R. (2023). Advancing pathogen genomics in resource-limited settings. Cell Genom.

[13] Roberts M.C., Holt K.E., Del Fiol G., Baccarelli A.A., Allen C.G. (2024). Precision public health in the era of genomics and big data. Nat Med.

[14] Urban L., Perlas A., Francino O. (2023). Real-time genomics for one health. Mol Syst Bio.

[15] Chiu C.Y., Miller S.A. (2019). Clinical metagenomics. Nat Rev Genet.

[16] Djordjevic S.P., Jarocki V.M., Seemann T. (2024). Genomic surveillance for antimicrobial resistance—a one health perspective. Nat Rev Genet.

[17] Keshaviah A., Diamond M.B., Wade M.J., Scarpino S.V., Global Wastewater Action Group (2023). Wastewater monitoring can anchor global disease surveillance systems. Lancet Glob Health.

[18] Oon Y.-L., Oon Y.-S., Ayaz M., Deng M., Li L., Song K. (2023). Waterborne pathogens detection technologies: advances, challenges, and future perspectives. Front Microbiol.

[19] van der Westhuizen H.-M., Soundararajan S., Berry T. (2024). A consensus statement on dual purpose pathogen surveillance systems: the always on approach. PLOS Glob Public Health.

[20] Armstrong G.L., MacCannell D.R., Taylor J. (2019). Pathogen genomics in public health. N Engl J Med.

[21] Collier S.A., Deng L., Adam E.A. (2021). Estimate of burden and direct healthcare cost of infectious waterborne disease in the United States. Emerg Infect Dis.

[22] ACDC National microbial genomics framework for public health 2025–2027. https://www.cdc.gov.au/resources/publications/national-microbial-genomics-framework-public-health-2025-2027.

[23] Webb J.R., Andersson P., Sim E. (2025). Implementing a national programme of pathogen genomics for public health: the Australian Pathogen Genomics Program (AusPathoGen). Lancet Microbe.

[24] NHMRC Australian Drinking Water Guidelines. https://www.nhmrc.gov.au/about-us/publications/australian-drinking-water-guidelines.

[25] AusTrakka AusTrakka. https://www.cdgn.org.au/austrakka.

[26] Hoang T., da Silva A.G., Jennison A.V., Williamson D.A., Howden B.P., Seemann T. (2022). AusTrakka: fast-tracking nationalized genomics surveillance in response to the COVID-19 pandemic. Nat Commun.

[27] Murphy H. (2024). https://www.abc.net.au/news/2024-11-11/gastro-cases-in-australia-are-surging/104562748.

[28] Western Australia Department of Health Health warning following rise in Cryptosporidiosis notifications. http://www.health.wa.gov.au/Media-releases/2025/March/Health-warning-following-rise-in-Cryptosporidiosis-notifications.

[29] Ho V. How worried should I be about cryptosporidiosis? Am I safe at the pool? The Conversation. https://theconversation.com/how-worried-should-i-be-about-cryptosporidiosis-am-i-safe-at-the-pool-223541.

[30] Ryan U., Lawler S., Reid S. (2017). Limiting swimming pool outbreaks of cryptosporidiosis–the roles of regulations, staff, patrons and research. J Water Health.

[31] NSW Health Cryptosporidiosis notifications in NSW residents. https://www1.health.nsw.gov.au/IDD/#/CRYPT/period.

[32] Queensland Health. Notifiable conditions annual reporting. https://www.health.qld.gov.au/clinical-practice/guidelines-procedures/diseases-infection/surveillance/reports/notifiable/annual.

[33] Victoria Department of Health Victoria, local public health areas and local government areas surveillance summary report. https://www.health.vic.gov.au/infectious-diseases/local-government-areas-surveillance-report.

[34] Western Australia Department of Health Statewide notifiable diseases weekly report. https://www.health.wa.gov.au/Articles/F_I/Infectious-disease-data/Statewide-notifiable-diseases-weekly-report.

[35] Australian Government DoH Disability and Ageing National Notifiable Diseases Surveillance System (NNDSS) fortnightly reports – 28 October to 10 November 2024. https://webarchive.nla.gov.au/awa/20250624110639/https://www.health.gov.au/resources/publications/national-notifiable-diseases-surveillance-system-nndss-fortnightly-reports-28-october-to-10-november-2024?language=en.

[36] Wood M., Simmonds L., MacAdam J., Hassard F., Jarvis P., Chalmers R.M. (2019). Role of filtration in managing the risk from *Cryptosporidium* in commercial swimming pools–a review. J Water Health.

[37] Cullinan L., McLean S., Dunn L. (2020). Preventing and controlling *Cryptosporidium* spp. in aquatic facilities: environmental health practitioners' experiences in Victoria, Australia. Aust N Z J Public Health.

[38] Lal A., Fearnley E., Wilford E. (2019). Local weather, flooding history and childhood diarrhoea caused by the parasite *Cryptosporidium* spp.: a systematic review and meta-analysis. Sci Total Environ.

[39] Ikiroma I.A., Pollock K.G. (2021). Influence of weather and climate on cryptosporidiosis—A review. Zoonoses Public Health.

[40] Lal A., Konings P. (2018). Beyond reasonable drought: hotspots reveal a link between the ‘big dry’and cryptosporidiosis in Australia's Murray Darling Basin. J Water Health.

[41] Williams S.V., Matthews E., Inns T. (2025). Retrospective case–case study investigation of a significant increase in *Cryptosporidium* spp. in England and Wales, August to September 2023. Euro Surveill.

[42] Elwin K., Hadfield S.J., Robinson G., Crouch N.D., Chalmers R.M. (2012). *Cryptosporidium viatorum* n. sp. (Apicomplexa: Cryptosporidiidae) among travellers returning to Great Britain from the Indian subcontinent 2007–2011. Int J Parasitol.

[43] Koehler A.V., Whipp M., Hogg G. (2014). First genetic analysis of *Cryptosporidium* from humans from Tasmania, and identification of a new genotype from a traveller to Bali. Electrophoresis.

[44] Tichkule S., Cacciò S.M., Robinson G. (2022). Global population genomics of two subspecies of *Cryptosporidium hominis* during 500 years of evolution. Mol Bio Evol.

[45] Bellinzona G., Nardi T., Castelli M. (2024). Comparative genomics of *Cryptosporidium parvum* reveals the emergence of an outbreak-associated population in Europe and its spread to the United States. Genome Res.

[46] CRU *Cryptosporidium* Reference Unit (CRU) public health Wales, Swansea. https://phw.nhs.wales/services-and-teams/cryptosporidium-reference-unit/.

[47] Chalmers R., Robinson G., Risby H. (2025). The transformation of a *Cryptosporidium* reference microbiology service to tackle the One Health challenge. Food Waterborne Parasitol.

[48] Morris A., Robinson G., Swain M.T., Chalmers R.M. (2019). Direct sequencing of *Cryptosporidium* in stool samples for public health. Front Public Health.

[49] Khan A., Alves-Ferreira E., Vogel H. (2024). Phylogenomic reconstruction of *Cryptosporidium* spp. captured directly from clinical samples reveals extensive genetic diversity. *bioRxiv*.

[50] NRCC National Reference Centre for cryptosporidiosis, Rouen. https://cnrcryptosporidioses.chu-rouen.fr/.

[51] Costa D., Razakandrainibe R., Basmaciyan L. (2022). A summary of cryptosporidiosis outbreaks reported in France and overseas departments, 2017–2020. Food Waterborne Parasitol.

[52] Costa D., Razakandrainibe R., Valot S. (2020). Epidemiology of cryptosporidiosis in France from 2017 to 2019. Microorganisms.

[53] Watier-Grillot S., Costa D., Petit C. (2022). Cryptosporidiosis outbreaks linked to the public water supply in a military camp, France. PLoS Negl Trop Dis.

[54] CDC CryptoNet. https://www.cdc.gov/cryptosporidium/php/cryptonet/index.html.

[55] Yoder J.S., Beach M.J. (2010). *Cryptosporidium* surveillance and risk factors in the United States. Exp Parasitol.

[56] Agyabeng-Dadzie F., Xiao R., Kissinger J.C. (2024). Genomics - current understanding, advances, and applications. Curr Trop Med Rep.

[57] Baptista R.P., Li Y., Sateriale A. (2022). Long-read assembly and comparative evidence-based reanalysis of *Cryptosporidium* genome sequences reveal expanded transporter repertoire and duplication of entire chromosome ends including subtelomeric regions. Genome Res.

[58] Baptista R.D.P., Xiao R., Li Y., Glenn T.C., Kissinger J.C. (2025). New T2T assembly of *Cryptosporidium parvum* IOWA II annotated with Legacy-Compatible Gene identifiers. Sci Data.

[59] Bayona-Vásquez N., Sullivan A., Beaudry M. (2024). Whole genome targeted enrichment and sequencing of human-infecting *Cryptosporidium* spp. Res Sq.

[60] Baptista R.P., Cooper G.W., Kissinger J.C. (2021). Challenges for *Cryptosporidium* population studies. Genes.

[61] Tichkule S., Jex A.R., Van Oosterhout C. (2021). Comparative genomics revealed adaptive admixture in *Cryptosporidium hominis* in Africa. Microb Genom.

[62] Bulumulla S., Ash A., Ryan U. (2026). Molecular analysis of *Cryptosporidium* species in Western Australian human populations (2023–2025), and the emergence of rare *C. hominis* IeA11G3T3 subtype. Parasitol Int.

[63] Koehler A.V., Wang T., Stevens M.A., Haydon S.R., Gasser R.B. (2025). Long-term molecular surveillance of *Cryptosporidium* and *Giardia* in wildlife in protected drinking water catchments. Parasit Vectors.

[64] Babineau M., Koehler A., Sait M. (2025). First comprehensive public health genotyping study of notifiable *Cryptosporidium* and *Giardia* infections in humans in southern Australia. J Clin Microbiol.

[65] Braima K., Harvie S., Trew I. (2022). Knowledge, attitude and practices towards *Cryptosporidium* among public swimming pool patrons and staff in Western Australia. Acta Parasitol.

[66] Braima K., Zahedi A., Oskam C. (2019). Retrospective analysis of *Cryptosporidium* species in Western Australian human populations (2015–2018), and emergence of the *C. hominis* IfA12G1R5 subtype. Infect Genet Evol.

[67] Ayres Hutter J., Dion R., Irace-Cima A. (2020). *Cryptosporidium* spp.: human incidence, molecular characterization and associated exposures in Québec, Canada (2016-2017). PLoS One.

[68] Guy R.A., Yanta C.A., Muchaal P.K. (2021). Molecular characterization of *Cryptosporidium* isolates from humans in Ontario, Canada. Parasit Vectors.

[69] EpiPulse European Centre for disease prevention and control. https://www.ecdc.europa.eu/en.

[70] Suominen K., Vainio A., Hokkanen P. (2025). Multilocus variable-number tandem-repeat analysis as an investigation tool in *Cryptosporidium parvum* outbreaks in Finland and Sweden in 2022. Microorganisms.

[71] Troell K., Stensvold C.R., Sannella A.R. (2025). Design, development, and testing of a new multi-locus sequence typing scheme for the zoonotic pathogen *Cryptosporidium parvum*. Curr Res Parasitol Vector Borne Dis.

[72] Risby H., Robinson G., Chandra N. (2023). Application of a new multi-locus variable number tandem repeat analysis (MLVA) scheme for the seasonal investigation of *Cryptosporidium parvum* cases in Wales and the northwest of England, spring 2022. Curr Res Parasitol Vector Borne Dis.

[73] Robinson G., Pérez-Cordón G., Hamilton C. (2022). Validation of a multilocus genotyping scheme for subtyping *Cryptosporidium parvum* for epidemiological purposes. Food Waterborne Parasitol.

[74] Robinson G., Chalmers R.M., Elwin K. (2025). Deciphering a cryptic minefield: a guide to *Cryptosporidium* gp60 subtyping. Curr Res Parasitol Vector Borne Dis.

[75] Gherasim A., Lebbad M., Insulander M. (2012). Two geographically separated food-borne outbreaks in Sweden linked by an unusual *Cryptosporidium parvum* subtype, October 2010. Euro Surveill.

[76] McCann R., Jones R., Snow J. (2014). An outbreak of cryptosporidiosis at a swimming club–can rapid field epidemiology limit the spread of illness?. Epidemiol Infect.

[77] Widerström M., Schönning C., Lilja M. (2014). Large outbreak of *Cryptosporidium hominis* infection transmitted through the public water supply, Sweden. Emerg Infect Dis.

[78] Goñi P., Almagro-Nievas D., Cieloszyk J., Lóbez S., Navarro-Marí J.M., Gutiérrez-Fernández J. (2015). Cryptosporidiosis outbreak in a child day-care center caused by an unusual *Cryptosporidium hominis* subtype. Enferm Infecc Microbiol Clín.

[79] McKerr C., Adak G.K., Nichols G. (2015). An outbreak of *Cryptosporidium parvum* across England & Scotland associated with consumption of fresh pre-cut salad leaves, May 2012. PLoS One.

[80] Utsi L., Smith S.J., Chalmers R.M., Padfield S. (2016). Cryptosporidiosis outbreak in visitors of a UK industry-compliant petting farm caused by a rare *Cryptosporidium parvum* subtype: a case-control study. Epidemiol Infect.

[81] Hall V., Taye A., Walsh B. (2017). A large outbreak of gastrointestinal illness at an open-water swimming event in the River Thames, London. Epidemiol Infect.

[82] Gopfert A., Chalmers R.M., Whittingham S. (2022). An outbreak of *Cryptosporidium parvum* linked to pasteurised milk from a vending machine in England: a descriptive study, March 2021. Epidemiol Infect.

[83] Hadfield S.J., Pachebat J.A., Swain M.T. (2015). Generation of whole genome sequences of new *Cryptosporidium hominis* and *Cryptosporidium parvum* isolates directly from stool samples. BMC Genom.

[84] Jones G., Matizanadzo J., Nelson A. (2025). A large *Cryptosporidium parvum* outbreak associated with a lamb-feeding event at a commercial farm in South Wales, March–April 2024: a retrospective cohort study. Epidemiol Infect.

[85] Peake L., Bardsley M., Bartram S. (2024). A large cryptosporidiosis outbreak associated with an animal contact event in England: a retrospective cohort study, 2023. Epidemiol Infect.

[86] Troell K., Hallström B., Divne A.-M. (2016). *Cryptosporidium* as a testbed for single cell genome characterization of unicellular eukaryotes. BMC Genom.

[87] Agyabeng-Dadzie F., Beaudry M.S., Deyanov A. (2025). Evaluating the benefits and limits of multiple displacement amplification with whole-genome Oxford nanopore sequencing. Mol Ecol Res.

[88] Corsi G.I., Tichkule S., Sannella A.R. (2023). Recent genetic exchanges and admixture shape the genome and population structure of the zoonotic pathogen *Cryptosporidium parvum*. Mol Ecol.

[89] Turner R., de Paula Baptista R., Rosenthal B.M., Kissinger J.C., Khan A. (2025).

[90] Zahedi A., Monis P., Aucote S. (2016). Zoonotic *Cryptosporidium* species in animals inhabiting Sydney water catchments. PLoS One.

[91] Barrosa A.D., Egan S., Feng Y., Xiao L., Ryan U. (2023). *Cryptosporidium* and *Giardia* in cats and dogs: what is the real zoonotic risk?. Curr Res Parasitol Vector Borne Dis.

[92] Barbosa A.D., Egan S., Feng Y., Xiao L., Ryan U. (2023). How significant are bats as potential carriers of zoonotic *Cryptosporidium* and *Giardia*?. Curr Res Parasitol Vector Borne Dis.

[93] Egan S., Barbosa A., Feng Y., Xiao L., Ryan U. (2024). Critters and contamination: zoonotic protozoans in urban rodents and water quality. Water Res.

[94] Egan S., Barbosa A.D., Feng Y., Xiao L., Ryan U. (2024). Rabbits as reservoirs: an updated perspective of the zoonotic risk from *Cryptosporidium* and *Giardia*. Vet Parasitol.

[95] Egan S., Barbosa A.D., Feng Y., Xiao L., Ryan U. (2024). Minimal zoonotic risk of cryptosporidiosis and giardiasis from frogs and reptiles. Eur J Protistol.

[96] Nolan M.J., Jex A.R., Koehler A.V., Haydon S.R., Stevens M.A., Gasser R.B. (2013). Molecular-based investigation of *Cryptosporidium* and *Giardia* from animals in water catchments in southeastern Australia. Water Res.

[97] Koehler A.V., Haydon S.R., Jex A.R., Gasser R.B. (2016). *Cryptosporidium* and *Giardia* taxa in faecal samples from animals in catchments supplying the city of Melbourne with drinking water (2011 to 2015). Parasit Vectors.

[98] Koehler A.V., Wang T., Haydon S.R., Gasser R.B. (2018). *Cryptosporidium viatorum* from the native Australian swamp rat *Rattus lutreolus* - an emerging zoonotic pathogen?. Int J Parasitol Parasites Wildl.

[99] Akande O.W., Carter L.L., Abubakar A. (2023). Strengthening pathogen genomic surveillance for health emergencies: insights from the World Health Organization's regional initiatives. Front Pub Health.

